# Neurobiological correlates and attenuated positive social intention attribution during laughter perception associated with degree of autistic traits

**DOI:** 10.1007/s00702-023-02599-5

**Published:** 2023-02-18

**Authors:** Anne Martinelli, Elgin Hoffmann, Carolin Brück, Benjamin Kreifelts, Thomas Ethofer, Dirk Wildgruber

**Affiliations:** 1 School of Psychology, Fresenius University of Applied Sciences, Marienburgstrasse 6, 60528 Frankfurt am Main, Germany; 2 Department of Psychiatry and Psychotherapy, Tübingen Center for Mental Health, University Hospital Tübingen, Calwerstrasse 14, 72076 Tübingen, Germany; 3 Department of Radiation Oncology, University Hospital Tübingen, Hoppe-Seyler-Str. 3, 72076 Tübingen, Germany; 4 Department of Biomedical Magnetic Resonance, University Hospital Tübingen, Otfried-Müller-Straße 51, 72076 Tübingen, Germany

**Keywords:** Autism spectrum disorder, Social intention attribution, Laughter processing, Temporal voice area, Fusiform face area

## Abstract

Laughter plays an important role in group formation, signaling social belongingness by indicating a positive or negative social intention towards the receiver. In adults without autism, the intention of laughter can be correctly differentiated without further contextual information. In autism spectrum disorder (ASD), however, differences in the perception and interpretation of social cues represent a key characteristic of the disorder. Studies suggest that these differences are associated with hypoactivation and altered connectivity among key nodes of the social perception network. How laughter, as a multimodal nonverbal social cue, is perceived and processed neurobiologically in association with autistic traits has not been assessed previously. We investigated differences in social intention attribution, neurobiological activation, and connectivity during audiovisual laughter perception in association with the degree of autistic traits in adults [*N* = 31, *M*_age_ (SD) = 30.7 (10.0) years, *n*_female_ = 14]. An attenuated tendency to attribute positive social intention to laughter was found with increasing autistic traits. Neurobiologically, autistic trait scores were associated with decreased activation in the right inferior frontal cortex during laughter perception and with attenuated connectivity between the bilateral fusiform face area with bilateral inferior and lateral frontal, superior temporal, mid-cingulate and inferior parietal cortices. Results support hypoactivity and hypoconnectivity during social cue processing with increasing ASD symptoms between socioemotional face processing nodes and higher-order multimodal processing regions related to emotion identification and attribution of social intention. Furthermore, results reflect the importance of specifically including signals of positive social intention in future studies in ASD.

**Supplementary Information:**

The online version contains supplementary material available at 10.1007/s00702-023-02599-5.

## Introduction

Laughter is a complex multimodal nonverbal social signal which can convey a wide range of social information. Laughter can signal a positive social intention towards the receiver, for example as a sign of interest and friendliness, thereby indicating a welcoming and inclusive attitude. Conversely, it may signal a negative social intention, indicating disliking or scorn, and thereby exclude others from the social group. In this way, laughter serves a pivotal role in the formation of groups and in maintaining social relationships (Davila Ross et al. 2009, 2010; Provine 2013). It is employed as a complex communicative tool in a variety of contexts, far beyond its role as a sign of amusement (Alter and Wildgruber 2019; Provine 2013, 2004; Vettin and Todt 2004). The utilization of laughter to communicate a wide spectrum of social intentions is associated with distinct variations in acoustic characteristics within the signal (Szameitat et al. 2009a, b). Studies indicate that these variations suffice for adult listeners to correctly and robustly identify a laugher’s social intention, based solely on the acoustic component of laughter sequences without further contextual information (Ritter et al. 2015; Szameitat et al. 2022; Szameitat et al. 2009a, b).

Given the importance the correct interpretation of laughter assumes in social contexts, it is of particular relevance to examine interindividual differences and difficulties in attributing social intention to laughter. Difficulties in the interpretation of social signals constitute a key characteristic of Autism Spectrum Disorder (ASD; APA 2013; WHO 1992). With respect to laughter, ASD has been associated with the fear of being laughed at, feeling unwell among laughing persons, and preferring to avoid situations in which one could be laughed at (Leader et al. 2018; Samson et al. 2011). These behavioral tendencies may reflect the uncertainties and obstacles people with ASD experience in the interpretation of laughter, as a complex social signal of acceptance or rejection. To our knowledge, previous studies have not assessed the association between autistic traits and such perceived social intention in others. Rather, the majority of experimental research has focused on the behavioral and neurobiological response in ASD to social stimuli in general (Chevallier et al. 2012; Clements et al. 2018) and to social exclusion in particular (Venturini and Parsons 2018). Previous findings indicate attenuated responses to social stimuli and specifically to social exclusion on both a behavioral (Andari et al. 2010; Silva et al. 2020) as well as a neurobiological (Masten et al. 2011; McPartland et al. 2011) level. This has been argued to lie in the differential processing of rejection experiences in accordance with reduced social motivation in ASD (Chevallier et al. 2012; Clements et al. 2018). However, outside of the laboratory, a preserved desire for social affiliation (Acker et al. 2018) and negative mental health outcomes associated with the lack of perceived social acceptance (Cage et al. 2018) are reported. Thus, it has been suggested that higher levels of experienced exclusion and ridicule in ASD (Little 2002; Tonnsen and Hahn 2016; Weiss and Fardella 2018) may operate via a conditioned fear response, leading to the development of a fear of being laughed at (gelotophobia; Leader et al. 2018; Samson et al. 2011). In this context, the current study examines whether autistic traits are associated with the differential perceived positive or negative social intention of laughter, and explores possible alterations in the associated neurobiological laughter processing.

The neurobiological correlates of social processing differences in ASD, for social cues other than laughter, have been the focus of extensive research. The current task-related neuroimaging literature presents an overarching picture characterized by neural hypoactivation during social tasks (e.g., face, speech and biological motion processing, identification and imitation, as well as mentalizing tasks) within central nodes of the social perception network. These include the prefrontal cortex, superior temporal gyrus and fusiform gyrus, along with the amygdala (Dichter 2012; Philip et al. 2012). Consistently, altered activation in the fusiform face area (FFA) during face processing (Critchley et al. 2000; Dalton et al. 2005; Domes et al. 2013; Scherf et al. 2015) and in the temporal voice area (TVA) during language and prosody processing (Eigsti et al. 2012; Gervais et al. 2004; Schelinski et al. 2016; Wang et al. 2006) have been reported. Differences in the activation of social brain regions are discussed as a consequence of reduced social experience and reduced preference for social stimuli, rather than as a primary dysfunction of these regions (Philip et al. 2012). Reduced experience with and preference for social stimuli is proposed to instead hamper perceptual skill development in processing e.g. face stimuli (Dichter 2012). Along with the task-related neural activation differences described above, reduced resting-state activation in the middle and superior temporal gyrus, precuneus, and posterior and mid-cingulate cortex has been reported in ASD, independently of social tasks or social function measures (Wang et al. 2018).

In addition to altered neural activation in ASD, a range of literature has explored connectivity measures for neurobiological correlates of social interaction differences in ASD (Ameis and Catani 2015; Aoki et al. 2017; Dimond et al. 2019; Hoffmann et al. 2016; Kana et al. 2011; Maximo et al. 2014). The implemented approaches span task-related and task-free functional connectivity as well as structural connectivity via a variety of white matter metrics. Here, too, numerous studies report hypoconnectivity among central nodes of the social perception network, including the FFA, the TVA, the mediofrontal cortex and the amygdala (Ameis and Catani 2015; Di Martino et al. 2014; Hoffmann et al. 2016; Welchew et al. 2005). These findings are furthermore associated with the degree of autistic symptoms in ASD, such as measured by the Social Responsiveness Scale (Gotts et al. 2012) or the social domains of the Autism Diagnostic Observation Schedule and Autism Diagnostic Interview-Revised (Supekar et al. 2013) and the Autism-Spectrum Quotient (Hoffmann et al. 2016). Finally, while hypoconnectivity is often reported among long-range connections (Ameis and Catani 2015; Aoki et al. 2017; Arnold Anteraper et al. 2019; Falahpour et al. 2016; Kana et al. 2011), increased connectivity has also been reported, for instance between the thalamus and sensory and motor cortices as well as sensory and subcortical networks (Cerliani et al. 2015; Conti et al. 2017; Fu et al. 2019, 2021; Pillai et al. 2018). Such hyperconnections have also been associated with the degree of autistic symptoms, both in the social (verbal and nonverbal communication) as well as in further (repetitive and restrictive behavior) domains (Maximo et al. 2014). Often, long-range hypoconnectivity is reported alongside local hyperconnectivity (Kana et al. 2011; Mash et al. 2018; Oldehinkel et al. 2019). EEG and MEG studies (O’Reilly et al. 2017), as well as brain development analyses across the lifespan (Courchesne et al. 2011; Courchesne and Pierce 2005; Hernandez et al. 2015; Nomi and Uddin 2015) corroborate the findings of concurrent hyper- and hypoconnectivity in ASD as contributing to both sensory and social-communicative differential processing in ASD via underlying network hierarchy imbalances (Dichter 2012; Hong et al. 2019; Philip et al. 2012).

Within the literature on the neurobiological correlates of laughter perception, assigning social intention to laughter has been associated with increased superior temporal, lateral parietal, and inferior and medial frontal cortex activation (Ethofer et al. 2020; Martinelli et al. 2019). However, the perception and neural processing of laughter as a multi-modal nonverbal social communication signal have not been studied in association with autistic traits. In line with the nature of autistic traits presenting along a spectrum of functionality, we sought to investigate differences in the intention attribution of laughter associated with increasing autistic trait scores, along with the associated neurobiological modulations in activation and connectivity during laughter perception and attribution. Based on self-report findings regarding the fear of being laughed at in autism (Leader et al. 2018; Samson et al. 2011), we expected more negative (less positive) laughter attributions with increasing autistic traits. Neurobiologically, we expected alterations in activation and connectivity during laughter processing within the social perception network, focusing on the FFA and TVA as central nodes in the face and voice processing, and on the amygdala in its central role in neurobiological accounts of ASD, as a saliency and self-relevance detector for social stimuli (Adolphs 2010; Sander et al. 2003; Zalla and Sperduti 2013). Finally, the perceived social intention of laughter can vary based on the perspective from which it is viewed. It has been demonstrated that viewing laughter as not being directed at oneself can decrease negative interpretation biases (e.g. negative social intention attribution) in social anxiety (Kreifelts et al. 2017). Thus, the current study will exploratively investigate whether the viewing perspective (directed at *versus* not directed at oneself) is differentially related to behavioral and neurobiological responses with increasing autistic traits. By investigating how laughter, as a highly relevant, nonverbal social cue, is differentially perceived and processed in association with the degree of autistic traits at the behavioral and neurobiological level, we seek to contribute to the current research on the neurobiological correlates for social communication differences related to ASD.

## Material and methods

### Participants

For purposes of including a wide variability in the degree of autistic traits among participants, recruitment was completed both via a special university hospital outpatient consultation for autism in adults as well as via e-mail communication with students and employees of the university. Via the outpatient consultation, all persons were contacted with a confirmed ASD diagnosis of high-functioning early childhood autism (*F*84.0) or Asperger-Syndrome (*F*84.5) who had previously consented to contact for study purposes. In total, 33 volunteers [*n*_female_ = 14, *M*_age_ (SD) = 31.3 (10.8) years] participated in the study. Two male participants without ASD were removed from analysis due to movement or technical problems (see Imaging Analysis) resulting in a total of *N* = 31 [*n*_female_ = 14, *M*_age_ (SD) = 30.7 (10.0)]. Of these, approximately one-third fulfilled the criteria for ASD diagnosis and had in the past been diagnosed by trained medical staff according to ICD-10 diagnostic criteria [*n* = 10, *n*_female_ = 2, *M*_age_ (SD) = 34.1 (11.1)]. Diagnoses were based on an assessment by two experienced clinicians and the completion and analysis of several diagnostic tests and questionnaires. This included a comprehensive anamnesis, evaluation of interaction behavior, a test of verbal intelligence (German MWT-B; Lehrl 2005), self-rating questionnaires including the Autism-Spectrum Quotient (AQ, see below) as well as questionnaires completed by close relatives reporting on childhood behavior: the Social Responsiveness Scale (Constantino et al. 2003), Social Communication Questionnaire (Rutter et al. 2003), and Marburg Rating Scale for Asperger’s Syndrome (Kamp-Becker et al. 2005). Among participants with an ASD diagnosis, five received medication (*n* = 1 each: Bupropion, Sertraline, Mirtazapine, combination Venlafaxine/Trimipramine and combination Methylphenidate, Promethazine, Agomelatine, Quetiapine). Furthermore, among participants with ASD, comorbid diagnoses included mild depressive episode (*n* = 4), moderate recurrent depressive disorder (*n* = 2), dysthymic disorder (*n* = 1) and obsessive–compulsive disorder (*n* = 1) as well as previous alcohol dependence and attention deficit disorder (*n* = 1 each). Among participants without an ASD diagnosis [*n* = 21, n_female_ = 12, *M*_age_ (SD) = 29.05 (9.3)], no past or present neurological or psychiatric disorders were reported according to the Structured Clinical Interview for DSM-IV (SCID-I; First et al. 1996, 2002).

All participants had successfully completed at least secondary school and had an IQ within the normal range (see Supplementary Table S1). Depressive symptom severity was assessed in all participants using the Beck Depression Inventory (BDI; Beck et al. 1996). The degree of autistic traits was determined using the Autism-Spectrum Quotient (AQ; Baron-Cohen et al. 2001), a self-report questionnaire covering the areas of social and communication skills, imagination, attention to detail and attention switching. Items are answered on a four-point scale indicating a degree of agreement with each item from “definitely disagree” to “definitely agree”. Scores were normalized according to the maximal attainable score, referred to as the *AQ percent score* or *autistic trait score*. Participants with and without ASD diagnoses differed significantly in AQ percent scores and BDI, and did not differ significantly in age, educational background or IQ (see Supplementary Table S1). All participants were native German speakers and right-handed according to the Edinburgh Inventory (Oldfield 1971).

This study was conducted in accordance with the Declaration of Helsinki’s ethical principles. The ethics committee of the University of Tübingen approved the study protocol prior to the start of the study. Participants were given comprehensive written information about the study aims and method and the opportunity to ask questions pertaining to details of the study, e.g. the experimental tasks and use of data. All participants gave written informed consent before taking part.

### Stimulus material and experimental task design

In a 3-Tesla MRI Scanner, participants were presented with two runs of sixty 1.5 s videos of male and female actors displaying short audiovisual sequences of laughter. Laughter sequences were elicited via self-induction, including scripting, imagination, emotional recall and body movement. Various laughter types were induced, based on a compilation of positive and negative social interaction scenarios. Examples include a relaxed, amusing or otherwise positive gathering with friends, being tickled by one’s partner, or observing a rival’s poor performance or mishap. After recording, laughter sequences were standardized in length and normalized for acoustic energy and head position; stimuli were furthermore selected based on at least average authenticity and recognizability ratings in independent healthy adult samples (for further details on stimulus production and pre-testing, see Kreifelts et al. 2014, 2017). Participants were instructed to rate each laughter sequence regarding the extent to which they perceived the laugher as signaling a positive (e.g. warm, friendly) social intention towards the receiver, indicated with the German word “Anlachen”. The opposite end of the scale represented laughter perceived as expressing a negative (e.g. excluding, belittling) social intention, indicated with the German word “Auslachen”. The four-point scale ranged from “strongly negative” to “slightly negative” to “slightly positive” to “strongly positive”.

Ratings were entered using a combined button-fiber optic system (LUMItouch, Photon Control, Burnaby, Canada) with four buttons pushed with the right hand. Participants were given 5 s after stimulus onset to press the button associated with their intention attribution. Following a variable intertrial interval between 3.0 and 5.0 s, the subsequent video was presented. On 10% of trials, a null event was inserted. During null events, the fixation cross remained on-screen for the length of the intertrial interval followed by an additional 10 s. This process reduces expectancy effects in the anticipation of the subsequent laughter stimulus. Furthermore, null events improve the baseline condition estimation by increasing the duration of the baseline (no stimulus) condition. This allows for a normalization of the hemodynamic response function, increasing the sensitivity of event-related study designs to detect main and differential effects (Friston et al. 1999; Josephs and Henson 1999). The order of laughter sequence presentation was randomized across participants. Laughter stimuli were presented using the software Presentation (Neurobehavioral Systems, Inc., Albany, CA, USA). The visual component of the stimuli was presented via back projection on a screen behind the MRI scanner and viewed over a mirror mounted on the head coil. The auditory component was played over MR-compatible headphones (MR confon GmbH, Magdeburg, Germany).

The task was completed in two runs in which participants were instructed to assume one of two observer perspectives. In the *self*-condition, participants were asked to imagine themselves as the target of the laughter. In the *other *condition, they were instructed to take the perspective of an uninvolved onlooker, watching another person laughing. The social intention of the laugher was to be rated out of this perspective. The sequence of task instruction (*self* or *other* first) and the horizontal orientation of the rating scale (positive or negative social intention on the left-hand side) were counter-balanced across participants.

### Image acquisition

Participants were scanned in a 3 T Siemens TRIO MRI Scanner (Siemens, Erlangen, Germany) with a 12-channel head coil. Functional images were acquired with an echo-planar imaging (EPI) sequence (TR = 1700 ms, TE = 30 ms, flip angle = 90°, 30 slices, 4 mm slice thickness and 1 mm gap, field of view (FoV) = 192 mm, voxel size 3 × 3 × 4 mm). A high-resolution structural image was acquired per participant using a magnetization-prepared rapid acquisition gradient echo sequence (TR = 2300 ms, TE = 2.96 ms, 176 slices, slice thickness = 1 mm, FoV = 256 mm). A static field map was acquired for off-line EPI-image distortion correction (TR = 400 ms, TE_1_ = 5.19 ms, TE_2_ = 7.65 ms, flip angle = 60°, 30 slices, slice thickness = 3 mm, no filter).

### Behavioral data analysis

Behavioral data were analyzed using SPSS Statistics Version 25 (IBM Corp., Armonk, NY, USA). Intention attributions were (re)coded such that 1 = *strongly negative* and 4 = *strongly positive* for all participants. According to the hypotheses, more negative/less positive attributions were expected with increasing autistic trait scores. For this purpose, an attribution bias was operationalized via two methods. First, to examine an overall shift across all participants towards more negative or away from positive social intention attributions with increasing autistic traits, a bivariate Pearson correlation was calculated between participants’ mean intention ratings and AQ percent scores. Second, attribution biases were operationalized per participant as the reduction in answer choices with increasing positivity of the attribution rating, calculated via regression coefficients (individual bias scores, see Supplementary behavioral methods). We hypothesized stronger reductions (steeper negative slopes) in answer choices from 1 (strongly negative) to 4 (strongly positive) with increasing AQ percent scores. Due to the presence of depressive disorder comorbidities in participants with ASD diagnoses, the relationship between attribution bias scores and AQ percent scores was furthermore assessed via partial correlations, controlling for depressive symptoms using the BDI. *Post-hoc* correlations between AQ percent score and answer choice selection within each answer category were calculated to elucidate whether a possible attribution bias is driven by a tendency to avoid answers of increasing positive intention, to prefer answers of increasing negative intention, or both. Supplementary between-group independent sample t tests were also calculated for differences in mean laughter rating, individual bias scores and answer choice selection across the answer categories between participants with and without ASD diagnoses (see Supplementary behavioral methods and results). Correlation coefficients were compared between *self* and *other* conditions to test for task specificity using Fisher-*Z* transformation.

For behavioral analyses, the necessity to include age and sex covariates was assessed using Pearson correlation coefficients (age) and independent-sample t-tests (sex). Answers with reaction times greater than 2 SD above or below the mean per participant were removed to prevent biasing due to responses to unattended stimuli.

### Image analysis

MRI image processing was completed using custom-written MATLAB R2019b scripts based on statistical parametric mapping software (SPM8; Wellcome Department of Imaging Neuroscience, London, UK; http://www.fil.ion.ucl.ac.uk/spm/). Image preprocessing consisted of: functional image realignment to the first image per session, image unwarping using a static field map, co-registration of the structural T1 MPRAGE to the functional image mean, segmentation to MNI standard space, spatial normalization and smoothing with an 8 mm FWHM Gaussian kernel. Two participants were removed from the analysis after preprocessing due to extensive movement or technical artifacts. Of the remaining participants, no movements greater than 3 mm between subsequent acquisition volumes or rotations over 0.1° of angle were observed.

First-level analysis was performed using an event regressor of 1.5 s duration for each of the 120 (two runs à 60) laughter stimuli convolved with the canonical hemodynamic response function. Six movement parameters and the mean signal were added as regressors of no interest to each model. Laughter sequence stimuli with reaction times more than 2 SD from the mean per participant were removed from the analysis. First-level contrasts were created for the (1) Main effect of laughter, (2) Attribution effect (rating-dependent parametric analysis), and (3) Task effect (*self* vs. *other* condition). For the Attribution effect, each laughter sequence regressor was parametrically weighted with the participant’s demeaned behavioral attribution rating for the respective sequence, with positive weights indicating more positive intention attributions and negative weights indicating more negative attributions. Second-level regression analyses were conducted with the AQ percent score as the predictor variable for each first-level contrast. Supplementary between-group independent sample t tests were also calculated for differences in laughter-related BOLD activation between participants with and without ASD diagnoses [see Supplementary neuroimaging methods and results (between-group analyses)]. Analyses were repeated including BDI scores to assess unique variance when controlling for depressive symptom differences between the groups.

### Psychophysiological interaction (PPI) analysis

PPI analyses were calculated to assess connectivity changes associated with ASD symptom scores for each first-level contrast above, namely (1) connectivity during overall laughter processing, (2) attribution-related connectivity, and (3) task-related connectivity. Seed regions were determined within three core socioemotional network nodes for audiovisual (face and voice) processing using localizer tasks for the bilateral temporal voice area (TVA) and fusiform face area (FFA), and the AAL anatomical atlas for the bilateral amygdala (AMY). Localizer tasks were performed per participant; the group results of the main effect of faces > [houses, objects, landscapes] (face localizer; Kanwisher et al. 1997) as well as voices > [environmental, animal sounds] (voice localizer; Belin et al. 2000) were saved as seeds (for detail on localizer tasks and seed determination see Supplementary functional connectivity analysis methods). The time course of mean BOLD response within this region was defined as the physiological term per participant.

Psychological terms were determined analog to the first-level GLM contrasts. *Overall laughter processing*: The psychological term consisted of + 1 at the onset of each laughter event for a total of 120 onsets. *Attribution-related:* Each laughter onset was weighted with the participant’s demeaned intention attribution rating for the respective laughter sequence. *Task-related*: The psychological term consisted of + 1 for each laughter onset within the *self* condition and − 1 for each laughter onset within the *other* condition. PPI terms were calculated as the product of the deconvolved physiological and psychological terms. The physiological, psychological and PPI terms were entered as regressors in first-level analyses. To determine autistic trait-related connectivity alterations, AQ percent score was entered as the predictor variable in three second-level multiple regression analyses, with the overall laughter, attribution-related, and task-related PPIs as the criterion variable, respectively.

Age and sex were included as covariates in all GLM and PPI analyses. Statistical significance was evaluated at voxel-level *p*_unc_ < 0.001 and cluster-level *p*_FWE_ < 0.05 to correct for multiple comparisons. Supplementary between-group independent sample t-tests were also calculated for differences in laughter-related connectivity between participants with and without ASD diagnoses [see Supplementary neuroimaging methods and results (between-group analyses)] and were repeated including BDI to control for depressive symptom differences between groups.

## Results

### Behavioral results

Behaviorally, age and sex were not associated with overall attribution ratings, answer choice regression coefficients or answer choice selection (age: all *r* < 0.27, all *p* > 0.156; sex: all *t* < 1.41, all *p* > 0.169). These were therefore not included as covariates for further behavioral analyses. The Pearson correlation between mean attribution rating per participant and AQ percent scores was not significant, while the reduction in answer choices with increasingly positive intention attributions (individual bias score) was significantly associated with AQ (see Table 1 and Supplementary behavioral results). This effect remained significant when controlling for BDI scores. The relationship between AQ percent score and answer choices per response category showed a significant reduction in the answer choice “strongly positive” with AQ, remaining significant in the between-group approach (see Supplementary behavioral results). A marginal increase in response choices in the category “slightly negative” with AQ scores was found, while the answer choices “strongly negative” and “slightly positive” showed no relationship to autistic trait score. No task effect was found, as the relationship between autistic trait scores and behavioral responses did not significantly differ in the *self* compared to the *other* condition.

**Table 1:** Behavioral results: correlation of AQ percent scores with mean social intention attributions and answer choice selection

	Mean laughter rating^a^	Individual bias score^b^	Level 1: strongly negative	Level 2: slightly negative	Level 3: slightly positive	Level 4: strongly positive
*r* (*p*)	− 0.21 (0.131)	− **0.35 (0.028)**	− 0.07 (0.360)	0.30 (0.050)	0.27 (0.072)	− **0.45 (0.006)**
*Z*_Fishers_ (*p*)^c^	− 0.46 (0.161)	− 0.65 (0.130)	0.29 (0.193)	0.40 (0.173)	0.43 (0.166)	− 1.12 (0.062)

Boldface print indicates significance below *α* < 0.05 (one-sided hypothesis testing)

^a^Correlation of mean laughter ratings and AQ percent scores across all participants

^b^Correlation between AQ percent scores and the individual bias score operationalized via regression coefficient: negative correlation indicates stronger decreases in answer selection with increasing positivity of the attribution with increasing autistic trait scores

^c^Fisher *Z* comparison of correlation coefficients between *self* and *other* condition

### Neuroimaging results

#### Relation between autistic trait score and neural activation during laughter processing

**Fig. 1:**
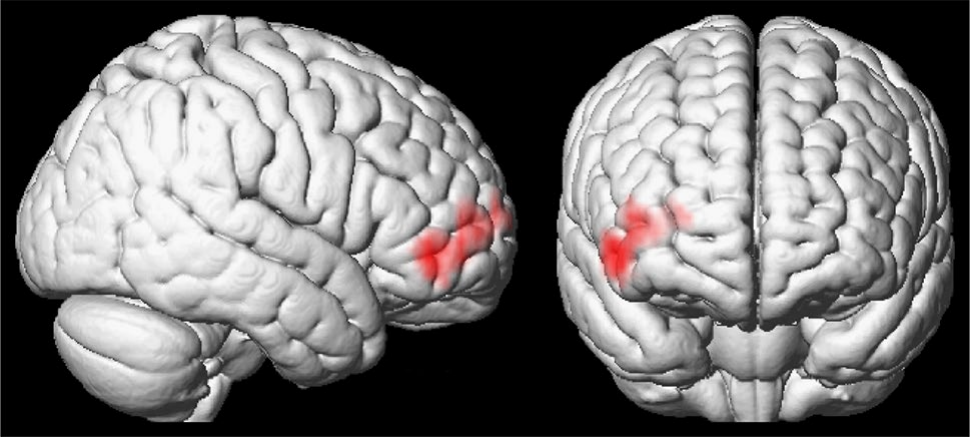
Association of BOLD response during laughter processing with AQ percent scores. A negative correlation was found between AQ percent scores and BOLD signal in the right inferior and middle frontal gyrus; i.e. activation during laughter processing in this cluster was reduced with increasing autistic traits. MNI peak coordinates: *x* = 42, *y* = 60, *z* = 6; *t*_peak_ = 5.44, *p*_FWE_; cluster size *k* = 156 voxels; *p*_FWE_ = 0.008. Whole-brain statistical thresholding at *p* < 0.001, uncorrected at voxel level; FWE corrected for multiple comparisons at cluster level *p* < 0.05

A negative association was found between BOLD response in the right inferior frontal gyrus (IFG) and middle frontal gyrus (MFG) and AQ percent score during laughter processing (see Fig. 1). A corresponding left hemispheric IFG/MFG cluster showed reduced activation in the ASD group in the between groups approach (see Supplementary neuroimaging results). No attribution or task-related effects were found in relation to AQ percent scores. Independently of AQ scores, more positive attributions were associated with increased BOLD signal in the ventral anterior cingulate cortex (see Supplementary neuroimaging results).

#### Connectivity alteration during laughter processing

**Fig. 2:**
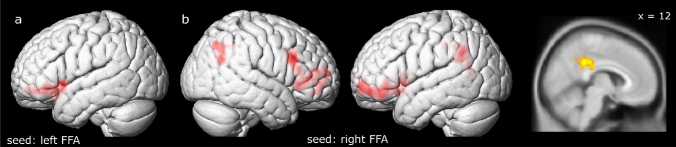
PPI functional connectivity analysis. Reduced connectivity during laughter processing with increasing autistic traits was found between **a** the left FFA and the left IFG, and **b** the right FFA and bilateral IFG and MFG, bilateral inferior parietal lobe, mid-cingulate cortex and left precuneus

During overall laughter processing, reduced connectivity was found with increasing autistic traits between the left FFA and left IFG (Fig. 2a and Table 2). Further reduced connectivity with increasing AQ scores was found from the right FFA to multiple brain regions, including bilateral IFG, bilateral inferior parietal lobe, mid-cingulate cortex, and precuneus (Fig. 2b and Table 2). Corresponding between-groups analyses showed reduced connectivity in the ASD group between the right FFA and bilateral IFG/MFG as well as bilateral inferior parietal lobe (see Supplementary neuroimaging results). Altered connectivity from the remaining seeds as well as attribution and task-related connectivity alterations in relation to the degree of autistic traits were not found.

**Table 2:** Reduced connectivity during laughter processing associated with autistic trait score

Seed	Anatomical peak location	Hemisphere	MNI coordinates			*t*-value	Cluster volume (voxels)	*p*_FWE_
			*x*	*y*	*z*			
Left FFA	Inferior frontal gyrus (IFG)	L	− 45	36	− 9	5.31	242	0.001
Right FFA	Inferior frontal gyrus (IFG)	L	− 48	39	− 12	6.79	372	< 0.001
	Inferior frontal gyrus (IFG)/middle frontal gyrus (MFG)	R	48	21	33	4.83	356	< 0.001
	Mid-cingulate gyrus	R	12	− 33	33	5.29	231	< 0.001
	Angular gyrus/inferior parietal lobe (IPL)	R	39	− 63	42	5.11	201	0.001
	Supramarginal gyrus/IPL	L	− 51	− 54	27	5.40	171	0.002
	Precuneus	L	− 12	− 57	33	5.40	87	0.045

Seeds: *FFA* fusiform face area

## Discussion

The current study investigated alterations in the perception and interpretation of laughter in relation to the degree of autistic traits in adults. Behavioral results indicate that, with increasing AQ scores, participants specifically declined to rate laughter as strongly positive, with a tendency to instead choose the answer “slightly negative” more often. Thereby, the current study does not find evidence of a negativity bias in the sense of more extreme negative intention interpretations (overall shift toward the negative end of the scale), nor of a general tendency for neutral or less extreme responses (more ambiguous responses closer to the middle of the response scale). Instead, a pattern specifically related to less identification of strongly positive social laughter emerged. Within the literature on social acceptance and rejection in ASD, previous studies have focused in particular on the behavioral and neural correlates of social exclusion (Venturini and Parsons 2018) and on more frequent experiences of social ridicule and exclusion in ASD (Weiss and Fardella 2018). It is possible that, as a consequence of such exclusion experiences, individuals with autistic traits develop a bias in the interpretation of social intention signals, and become hesitant or uncertain specifically in attributing positive social intention, such as welcoming laughter, as observed in the current study. This relationship remained when including depressive symptoms as a covariate, suggesting the process to be independent of depressive disorder comorbidity in ASD.

Neurobiologically, laughter processing was associated with less activation of the right MFG and IFG with increasing autistic trait scores. Furthermore, reduced functional connectivity was found between the left and right FFA with frontal, cingulate as well as parietal regions involved in social information processing, including the MFG and IFG, the inferior parietal cortex and the mid-cingulate gyrus. These results replicate previous findings of hypoactivity and hypoconnectivity in ASD during social cue processing (Dichter 2012; Hoffmann et al. 2016; Philip et al. 2012), in particular in connections from lower-order sensory processing to higher-order association regions involved in executive and attentional control and making inferences about the mental states of others (Lynn et al. 2018; Maximo et al. 2014; Wolff et al. 2013). Furthermore, at least one early study on top-down modulation during an attention-to-faces task (Bird et al. 2006) has reported decreased attentional modulation of face-selective regions (left fusiform gyrus) in ASD. Finally, reduced connectivity was found in the mid-cingulate gyrus, an area showing reduced activity (Wang et al. 2018) and connectivity (Lau et al. 2019) in ASD during resting state. The current results thereby show evidence of reduced recruitment of social processing nodes known to be less responsive in ASD, both within and independently of a social context. Thus, these alterations are likely to represent independent, rather than laughter specific, processing differences, which may underlie a wide spectrum of social interaction difficulties in ASD.

In the current study, hypoactivation was found during a social cue processing task requiring decisions regarding the mental state and intention of a social interaction partner. Previous studies have implicated the lateral frontoparietal regions showing reduced FFA connectivity in the current study to the attribution of social acceptance and experiencing social inclusion as well as social cue perception after social inclusion (Bolling et al. 2012, 2013; Ethofer et al. 2020). Furthermore, one of the few previous studies in ASD involving social affiliation attributions (“friend” or “foe” judgments, Watanabe et al. 2012) reported reduced recruitment among these and further medial and lateral (frontal) structures (IFG, anterior cingulate cortex (ACC), ventro- and dorsomedial prefrontal cortex (vm/dmPFC), bilateral insula). Reduced activation within these nodes predicted ASD communication differences. Finally, while not yet investigated in ASD, evidence of altered prefrontal-posterior functional coupling during positive laughter perception and decreased vmPFC, vACC and insula activation during humorous stimulus perception has been reported in individuals with gelotophobia (Chan 2016; Papousek et al. 2016). These findings have been interpreted as a reduced impact of signals of joy and humor on the individual. Thus, although the current results do not demonstrate direct neural correlates of decreased socially inclusive attributions in response to laughter, the observed neurobiological patterns should be further investigated to determine the relationship to both the perception of positive laughter and more generally to positive social nonverbal stimuli in ASD.

### Limitations

It should be noted that the current study includes a relatively small sample of individuals both fulfilling and not fulfilling the clinical criteria for ASD. Furthermore, participants with and without ASD diagnoses differed not only in AQ scores but also in medication and in depressive symptoms. While the variability of AQ scores shows an even continuation across a wide range, allowing for dimensional analyses in line with a spectrum understanding of autistic trait presentation and functionality, these results should be replicated in larger samples including more participants with a clinical diagnosis of ASD as well as different types of nonverbal socially inclusive cues. Moreover, psychopharmacological medication as well as depressive symptoms should be considered as possible confounding variables. While the patterns of results remained consistent when controlling for BDI scores, the effect of the psychopharmacological medication is more difficult to control. For the behavioral data, the correlation between AQ scores and attribution bias scores remained marginally significant, albeit underpowered, when evaluated within the unmedicated participants without ASD (Supplementary behavioral results). These findings thereby provide some evidence for the independence of the relationship from medication use.

The current study design does not allow exploration of whether the observed behavioral and neural processing parameters related to autistic traits are specific for laughter, nor to what extent these can be explained by the individual unimodal acoustic or visual (face) processing components. Furthermore, due to existent differences in experienced bullying and social exclusion in ASD, including increased experiences of being laughed at, it is not possible with the current design to distinguish differences in social intention attributions related to perceptual processing versus conditioned learning (Bolling et al. 2015; Grennan et al. 2018) or an interaction of the two. Finally, reduced neural responses and connectivity during laughter processing were not linearly related to behavioral intention attributions in the current study. Further investigation will be necessary to determine whether expectancy effects may be involved, driving non-linear relationships between the neural response to laughter and the social intention attributed to each stimulus (e.g. Ethofer et al. 2020).

### Implications

The current results provide the first findings of the neurobiological correlates of laughter processing in relation to autistic traits in adults. Decreased frontal activation and decreased connectivity between face-sensitive processing areas and higher-order frontal and parietal regions may underlie differences in laughter processing in ASD. Attributions of positive social intention in others are specifically reduced, which may make social inclusion cues particularly difficult to interpret in individuals with increasing degrees of autistic traits. The current study brings awareness to the limited literature on autism-related differences in the perception and neurobiological processing of inclusive, positive social signals such as laughter. Analogous to developments in other psychopathological domains (e.g., depression and bipolar disorder, De Panfilis et al. 2015; Malejko et al. 2018, 2019; Zhang et al. 2017), and in light of the health benefits specifically related to social inclusion in ASD (Cage et al. 2018; McConkey et al. 2020), the behavioral and neurobiological processing and interpretation of signs of social inclusion in ASD warrant more careful examination in future research.

## Supplementary Information

Below is the link to the electronic supplementary material.

Supplementary file1 (PDF 305 KB)

## Data Availability

The datasets generated and/or analysed during the current study are available from the corresponding author on reasonable request.
