# Supplementary material for: Neurobiological correlates and attenuated positive social intention attribution during laughter perception associated with degree of autistic traits
Source: J Neural Transm (Vienna). 2023 Feb 18;130(4):585–96. doi: 10.1007/s00702-023-02599-5 (PMC10049931; doi:10.1007/s00702-023-02599-5)
Supplement: Supplementary file 1 — Supplementary file1 (PDF 305 KB) [file 702_2023_2599_MOESM1_ESM.pdf]

Supplementary Material for:

Neurobiological correlates and attenuated positive social intention attribution during laughter perception associated with degree of autistic traits

Martinelli, A. [a*], Hoffmann, E. [b,c], Brück, C. [b], Kreifelts, B. [b], Ethofer, T [b,d], & Wildgruber, D.[b]

[a] School of Psychology, Fresenius University of Applied Sciences, Marienburgstrasse 6, 60528 Frankfurt am Main, Germany

[b] Department of Psychiatry and Psychotherapy, Tübingen Center for Mental Health, University Hospital Tübingen, Calwerstrasse 14, 72076 Tübingen, Germany

[c] Department of Radiation Oncology, University Hospital Tübingen, Hoppe-Seyler-Str. 3, 72076 Tübingen, Germany

[d] Department of Biomedical Magnetic Resonance, University Hospital Tübingen, Otfried-Müller-Straße 51, 72076 Tübingen, Germany

*** Corresponding author:** Anne Martinelli, anne.martinelli@hs-fresenius.de

Supplementary participant information

Table S1 Participant characteristics by diagnostic status

Group<br>N (female:male)	With ASD<br>10 (2:8)		Without ASD<br>21 (12:9)		
	M	***SD***	***M***	***SD***	***p***
**Age (years)**	34.1	11.1	29.0	9.3	.193
**AQ percent**	74.0	18.5	19.3	14.6	<.001
**BDI**	8.9	4.7	2.4	1.9	.003
**MWT-B IQ**	118	17.9	118	19.5	.977

Notes: p-values are given for the corresponding independent sample t-test per variable between groups; AQ percent = percent of maximum attainable score on the Autism-Spectrum Quotient (Baron-Cohen et al., 2001). BDI = Beck Depression Inventory (Beck et al., 1996); MWT-B IQ = calculated IQ value from the Mehrfachwahl-Wortschatz-Intelligenztest (Lehrl, 2005).

Supplementary behavioral methods and results

Attributional biases were operationalized over all participants as the mean individual laughter intention attribution rating correlated with AQ score (Baron-Cohen et al., 2001). Furthermore, an individual bias score was calculated per participant as the regression coefficient representing the change (slope) in answer choice selection with increasing positivity of answer choices from 1 (strongly negative) to 4 (strongly positive). Assuming no answering bias towards or away from more positive or more negative answer choices, a participant's regression coefficient would not differ from zero. The association of answering tendencies with AQ was calculated by correlating participants' regression coefficients with AQ score. Given systematic answering tendencies, such that fewer answer choices with increasing positivity are made, or answer choices of increasing negativity are chosen more often, less positive / more negative slopes would be expected with increasing AQ.

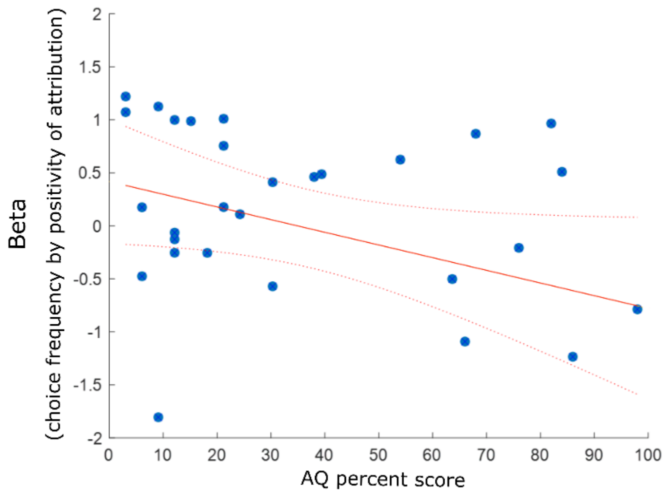

Fig. S1 Bias of social intention attributions and AQ percent scores Attribution biases operationalized per participant as the slope of change in answer choice frequency with increasing positivity of attributions. More negative regression coefficients indicate lower endorsement of increasingly positive intention attributions. Scatter plot depicts less positive / more negative regression coefficients (y-axis) with increasing AQ scores (x-axis), indicating that with higher autistic trait scores, the reduction in answer choice selection corresponding to more positive attributions became stronger (larger negative slope).

The relationship between individual bias scores and AQ percent scores was significant (Table 1: r = -.35, p = .028, Figure S1), and remained significant when controlling for depressive symptoms (BDI scores) via partial correlation (r = -.35, p = .033). Taking a group approach, comparing participants with and without ASD diagnosis, an independent sample t-test did not show significant group differences in the mean standardized regression coefficient (mean ASD = -0.30; mean without ASD = 0.11; t(29) = 1.01, p = .160). Finally, the correlation between regression coefficients and AQ percent score showed a marginally significant negative correlation of medium effect size both within the ASD diagnosis group (r = -.46, p = .092) as well as in the group without ASD (r = -.31, p = .087). Answer choice selection for the category "strongly positive" differed significantly between groups (mean ASD = 25.7% answer choices; mean without ASD = 16.6% answer choices; t(29) = 2.09, p = .023), while answer choice selection in the remaining answer categories were not significantly different (all t(29) < 1.17, all p > .312).

Supplementary functional connectivity analysis methods

Localizer Tasks for Psychophysiological Interaction (PPI) Seed Determination

Localizer tasks were performed to determine the peak face sensitive region within the bilateral fusiform gyrus (seed for the fusiform face area, FFA) as well as the peak voice sensitive region within the bilateral superior temporal gyrus (seed for the temporal voice area, TVA). Both localizer tasks were based on established experimental paradigms for face and voice sensitive area determination (voice localizier: Belin et al., 2000; face localizer: Kanwisher et al., 1997, adaptation see Hoffmann et al., 2016; Schwarz et al., 2019)

In the face localizer task, eight 30-second blocks of 45 static pictures were shown. Pictures were presented block-wise from each of the following four categories: human faces, everyday objects, landscapes and houses (Kanwisher et al., 1997). Each category was presented in two blocks. Blocks were separated by an inter-block interval of 20 seconds, in which a fixation cross was presented in the middle of the screen. Participants completed a one-back task to assure attention. Thereby, a button was to be pressed with the right index finger when the presented picture was identical to the preceding picture. Four regressors of interest were defined, one each for faces, objects, landscapes and houses. The contrast image Faces > (Houses, Objects, Landscapes) was calculated to identify face sensitive regions. The resulting group-level bilateral clusters situated within the fusiform gyrus were extracted as masks for the FFA seeds.

The voice localizer task implemented 24 blocks of sounds from the following three categories: 12 blocks of human vocal sounds, including speech, cries and laughter, 6 blocks of environmental sounds such as screeching tires or church bells, and 6 blocks of animal sounds such as mooing or galloping (Belin et al., 2000). In addition, 12 blocks of eight-second silence were presented. Blocks were randomized with the restriction that two blocks of silence were not presented sequentially. Participants conducted this task with eyes closed as a passive listening task. Three regressors were defined for human vocal sounds, environmental and animal sounds. The contrast Voices > (Environmental Sounds, Animal Sounds) was calculated to identify voice sensitive regions. The resulting group-level bilateral clusters situated on the superior temporal gyrus were extracted as masks for the TVA seeds.

Finally, as a central node of socioemotional saliency recognition, masks for the bilateral amygdalae were extracted from the automated anatomical labeling atlas (AAL) implemented in xjView SPM 8 toolbox (Tzourio-Mazoyer et al., 2002). The six final seed regions are shown in Supplementary Figure S2 and the localizer-based seeds are defined in Supplementary Table S2.

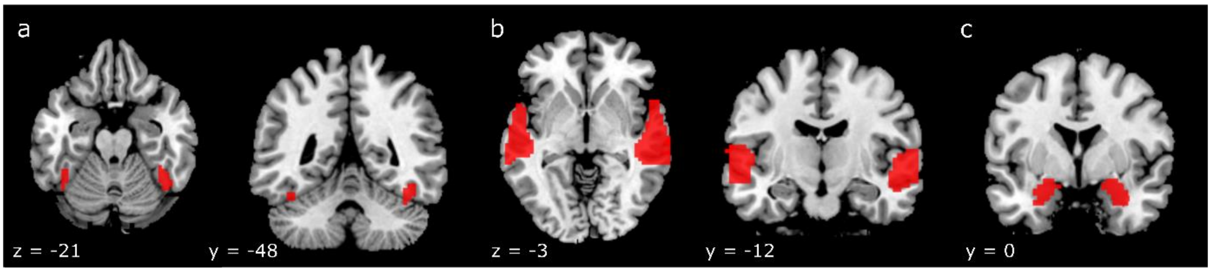

Fig. S2 PPI-seeds (a) FFA-seed determination using the group contrast of the face localizer task (Faces > Houses, Objects, Landscapes) within the fusiform gyrus. (b) TVA-seed determination using the group contrast of the voice localizer task (Voices > Environmental Sounds, Animal Sounds) within the superior temporal gyrus. (c) Amygdala-seed determination using a bilateral amygdala mask based on the AAL atlas (xjView SPM 8 toolbox; https://www.alivelearn.net/xjview).

Table S2 PPI-seed peak voxel coordinates determined using localizer tasks

Seed	Hemi-sphere	MNI coordinates			*t*-value	Volume (voxels)
		x	*Y*	*z*		
Fusiform Face Area	R	42	-48	-24	7.62	74
	L	-42	-57	-21	4.62	26
Temporal Voice Area	R	60	-9	-3	10.14	941
	L	-63	-12	-3	11.15	872

Supplementary neuroimaging methods and results (between-group analyses)

Methods: In addition to the fully dimensional approach according to the spectral characterization of ASD, a supplemental group comparison was also calculated. Hereby, an independent sample t-test was performed between the groups with (*n* = 10) and without (*n* = 21) a clinical ASD diagnosis. As in the main analysis, age and sex were controlled for as covariates of no interest. The between-groups comparison was performed for possible differences during neural activation (GLM-approach) as well as in connectivity (PPI-approach) during laughter processing.

Results: Group differences in GLM and PPI are shown in Figure S3 and reported in Table S3. Participants with ASD showed reduced activation within the left inferior frontal gyrus (IFG) during laughter processing compared to participants without ASD (Figure S3a). Reduced connectivity between the right FFA and bilateral IFG as well as bilateral supramarginal / angular gyrus and inferior parietal lobe was found in participants with compared to those without ASD (Figures S3b). The pattern of results did not change when including BDI as an additional covariate of no interest, and BDI alone did not explain any unique variance in the neural response.

Discussion: In the overall laughter processing activation, both the dimensional and group-based approaches showed subthreshold bilateral IFG activation differences dependent on AQ percent score or ASD diagnostic status. In the dimensional approach, the reduced activation with increasing AQ scores in the left IFG cluster remained significant after voxel and cluster-wise thresholding, while in the group comparison, the right IFG cluster remained significant after thresholding. Regarding connectivity, reduced right FFA to frontal and parietal cortical regions in ASD remained stable in the group comparison. Thus, the group analyses largely confirm the findings of the dimensional analyses. Overall, these data provide evidence of linear neural correlates of laughter processing spanning a continuum of autistic traits from the nonclinical to clinical domain, independently of depressive symptom severity.

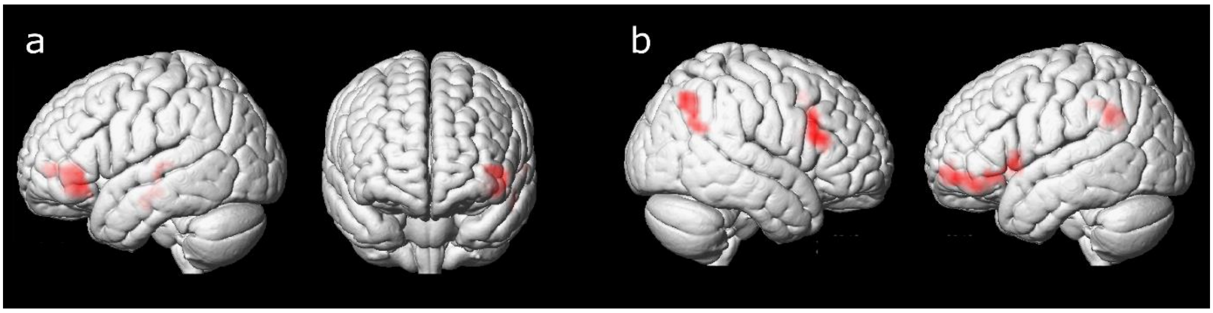

Fig. S3 Group differences in neural activation and connectivity during laughter processing. (a) Reduced BOLD response during laughter processing in ASD in left inferior frontal gyrus. (b) Reduced connectivity during laughter processing in ASD between the right FFA (seed) and bilateral inferior and middle frontal gyrus and bilateral inferior parietal lobe. Whole-brain statistical thresholding at $p < .001$, uncorrected at voxel level; FWE corrected for multiple comparisons at cluster level $p < .05$.

Table S3 Group differences in activation and connectivity during laughter processing

Anatomical peak location	Hemi-sphere	MNI coordinates			*t*-value	Cluster volume (voxels)	p_{FWE}
		x	y	z			
Activation (GLM)							
Inferior frontal gyrus	L	-45	36	-15	5.90	215	.001
Connectivity (PPI Seed: right FFA)							
Inferior frontal gyrus / Middle frontal gyrus	L	-48	42	-12	6.70	285	<.001
Inferior frontal gyrus / Middle frontal gyrus	R	63	27	18	4.11	188	.001
Angular gyrus / Inferior parietal lobe (IPL)	R	42	-63	42	4.44	199	.001
Supramarginal gyrus / IPL	L	-54	-51	36	4.19	105	.024

Notes: All results represent reduced BOLD response and reduced connectivity in participants with ASD compared to participants without ASD. FFA = fusiform face area. GLM = General linear model, BOLD-based activation approach; PPI = Psychophysiological interaction-based connectivity approach.

Supplementary neuroimaging methods and results (AQ-independent analyses)

Methods: In addition to the AQ-dependent analyses, two supplementary, AQ-independent whole-brain analyses were calculated. The purpose of these analyses was to explore overall attribution and task-related BOLD associations independently of autistic trait level. To this end, the first-level contrasts created for the attribution effect (rating-dependent parametric analysis) and task effect (*self* vs. *other* condition) were used. For the attribution effect, each laughter sequence regressor was parametrically weighted with the participant's demeaned behavioral attribution rating for the respective sequence, with positive weights indicating more positive intention attributions and negative weights indicating more negative attributions. Second level analyses (one-sample t-tests) were conducted on the group level for attribution and task-related contrasts.

Results: AQ-independent activation patterns yielded a significant result related to attribution ratings. More positive intent attributions were associated across all participants with an increase in activation in the ventral anterior cingulate cortex (vACC) during laughter processing (Figure S4). No task-related AQ-independent BOLD responses were found.

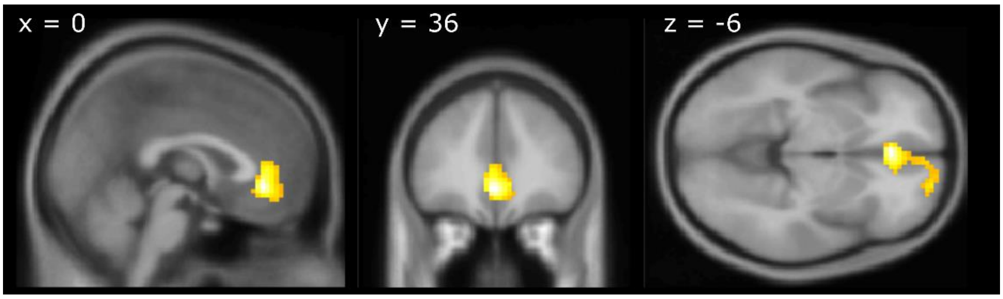

Fig. S4 Autistic trait-independent attribution effect BOLD response in the ventral anterior cingulate cortex during laughter processing was associated with more positive attribution ratings across all participants. This effect was independent of autistic trait score. MNI peak coordinates: x = 0, y = 36, z = -6; t_{peak} = 6.38, p_{FWE} < .001; cluster size k = 249 voxels. Whole-brain statistical thresholding at p < .001, uncorrected at voxel level; FWE corrected for multiple comparisons at cluster level p < .05.

Discussion: Among all participants, attributions of increasing positive social intention were associated with greater vACC activation. Within the limited literature on the neural correlates of social acceptance, associated neural processing has been localized among lateral frontoparietal regions (Bolling et al., 2013, 2012; Ethofer et al., 2020). Medial structures including the medial prefrontal (mPFC) and (dorsal) anterior and posterior cingulate (dACC/PCC) cortices, in contrast, are more commonly associated with social pain, activated in paradigms of social exclusion or rejection (Eisenberger and Lieberman, 2003; Vijayakumar et al., 2017; Williams and Jarvis, 2006) as well as during perceived socially excluding laughter (Ethofer et al., 2020). The ventral ACC has been implicated less often in such paradigms, yet has been (negatively) associated with attenuated social pain following social support (Onoda et al., 2009), and with both exclusion as well as fairplay, relative to control, conditions (Bolling et al., 2011). In the discussion surrounding the conceptualisation of the (d)ACC as part of a neural sociometer (Eisenberger et al., 2011), ever more studies report ACC responding to social evaluation and (particularly unexpected) social involvement in both an inclusive (positive) as well as an exclusive (negative) direction (Cheng et al., 2020; Dalgleish et al., 2017; Perini et al., 2018). The current results indicate that this broader, valence independent, processing of perceived self-referential judgements and social saliency may extend ventrally within the cingulate cortex, in particular when active attributional processes regarding the social intention of the social counterpart are involved.

References

Baron-Cohen, S., Wheelwright, S., Skinner, R., Martin, J., Clubley, E., 2001. The Autism-Spectrum Quotient (AQ): Evidence from Asperger Syndrome/High-Functioning Autism, Males and Females, Scientists and Mathematicians. J. Autism Dev. Disord. 31. https://doi.org/10.1023/A:1005653411471

Beck, A.T., Steer, R.A., Ball, R., Ranieri, W.F., 1996. Comparison of Beck depression inventories -IA and -II in psychiatric outpatients. J. Pers. Assess. 67. https://doi.org/10.1207/s15327752jpa6703_13

Belin, P., Zatorre, R.J., Lafaille, P., Ahad, P., Pike, B., 2000. Voice-selective areas in human auditory cortex. Nature 403, 309–312. https://doi.org/10.1038/35002078

Bolling, D.Z., Pelphrey, K.A., Kaiser, M.D., 2013. Social inclusion enhances biological motion processing: A functional near-infrared spectroscopy study. Brain Topogr. 26, 315–325. https://doi.org/10.1007/s10548-012-0253-y

Bolling, D.Z., Pelphrey, K.A., Vander Wyk, B.C., 2012. Differential brain responses to social exclusion by one's own versus opposite gender peers. Soc. Neurosci. 7, 331–346. https://doi.org/10.1080/17470919.2011.623181.Differential

Bolling, D.Z., Pitskel, N.B., Deen, B., Crowley, M.J., McPartland, J.C., Mayes, L.C., Pelphrey, K.A., 2011. Dissociable Brain Mechanisms for Processing Social Exclusion and Rule Violation. Neuroimage 54, 2462–2471. https://doi.org/10.1016/j.neuroimage.2010.10.049.Dissociable

Cheng, T.W., Vijayakumar, N., Flournoy, J.C., Op de Macks, Z., Peake, S.J., Flannery, J.E., Mobasser, A., Alberti, S.L., Fisher, P.A., Pfeifer, J.H., 2020. Feeling left out or just surprised? Neural correlates of social exclusion and overinclusion in adolescence. Cogn. Affect. Behav. Neurosci. 20, 340–355. https://doi.org/10.3758/s13415-020-00772-x

Dalgleish, T., Walsh, N.D., Mobbs, D., Schweizer, S., van Harmelen, A.-L., Dunn, B., Dunn, V., Goodyer, I., Stretton, J., 2017. Social pain and social gain in the adolescent brain: A common neural circuitry underlying both positive and negative social evaluation. Sci. Rep. 7, 42010. https://doi.org/10.1038/srep42010

Eisenberger, N.I., Inagaki, T.K., Muscatell, K.A., Byrne Haltom, K.E., Leary, M.R., 2011. The Neural Sociometer: Brain Mechanisms Underlying State Self-esteem. J. Cogn. Neurosci. 23, 3448–3455. https://doi.org/10.1162/jocn_a_00027

Eisenberger, N.I., Lieberman, M.D., 2003. Does Rejection Hurt? An fMRI Study of Social Exclusion. Sci. Reports 302, 290–292.

Ethofer, T., Stegmaier, S., Koch, K., Reinl, M., Kreifelts, B., Schwarz, L., Erb, M., Scheffler, K., Wildgruber, D., 2020. Are you laughing at me? Neural correlates of social intent attribution to auditory and visual laughter. Hum. Brain Mapp. 41, 353–361. https://doi.org/10.1002/hbm.24806

Hoffmann, E., Brueck, C., Kreifelts, B., Ethofer, T., Wildgruber, D., 2016. Reduced functional connectivity to the frontal cortex during processing of social cues in autism spectrum disorder. J. Neural Transm. 123, 937–947. https://doi.org/10.1007/s00702-016-1544-3

Kanwisher, N., McDermott, J., Chun, M.M., 1997. The Fusiform Face Area: A Module in Human Extrastriate Cortex Specialized for Face Perception. J. Neurosci. 17, 4302–4311. https://doi.org/10.1109/CDC.2005.1583375

Lehrl, S., 2005. Mehrfachwahl-Wortschatz-Intelligenztest MWT-B, 5. unverän. ed. Spitta Verlag, Balingen.

Onoda, K., Okamoto, Y., Nakashima, K., Nittono, H., Ura, M., Yamawaki, S., 2009. Decreased ventral anterior cingulate cortex activity is associated with reduced social pain during emotional support. Soc. Neurosci. 4, 443–454. https://doi.org/10.1080/17470910902955884

Perini, I., Gustafsson, P.A., Hamilton, J.P., Kämpe, R., Zetterqvist, M., Heilig, M., 2018. The salience of self, not social pain, is encoded by dorsal anterior cingulate and insula. Sci. Rep. 8, 1–9. https://doi.org/10.1038/s41598-018-24658-8

Schwarz, L., Kreifelts, B., Wildgruber, D., Erb, M., Scheffler, K., Ethofer, T., 2019. Properties of face localizer activations and their application in functional magnetic resonance imaging (fMRI) fingerprinting. PLoS One 14, 1–21. https://doi.org/10.1371/journal.pone.0214997

Tzourio-Mazoyer, N., Landeau, B., Papathanassiou, D., Crivello, F., Etard, O., Delcroix, N., Mazoyer, B., Joliot, M., 2002. Automated anatomical labeling of activations in SPM using a macroscopic anatomical parcellation of the MNI MRI single-subject brain. Neuroimage 15. https://doi.org/10.1006/nimg.2001.0978

Vijayakumar, N., Cheng, T.W., Pfeifer, J.H., 2017. Neural correlates of social exclusion across ages: A coordinate-based meta-analysis of functional MRI studies. Neuroimage. https://doi.org/10.1016/j.neuroimage.2017.02.050

Williams, K.D., Jarvis, B., 2006. Cyberball: A program for use in research on interpersonal ostracism and acceptance. Behav. Res. Methods 38, 174–180.
